# Dual-band bound states in the continuum based on hybridization of surface lattice resonances

**DOI:** 10.1515/nanoph-2022-0427

**Published:** 2022-11-01

**Authors:** Xiang Du, Lei Xiong, Xueqian Zhao, Shuai Chen, Jianping Shi, Guangyuan Li

**Affiliations:** College of Physics and Electronic Information, Anhui Normal University, Wuhu 241000, China; CAS Key Laboratory of Human-Machine Intelligence-Synergy Systems, Shenzhen Institute of Advanced Technology, Chinese Academy of Sciences, Shenzhen 518055, China; SIAT Branch, Shenzhen Institute of Artificial Intelligence and Robotics for Society, Shenzhen 518055, China xiang.du@siat.ac.cn; School of Information Science and Engineering, Yunnan University, Kunming 650500, China lei.xiong@siat.ac.cn; School of Computer, Electronics and Information, Guangxi University, Nanning 530004, China xq.zhao@siat.ac.cn; Tianjin H-Chip Technology Group Corporation, Tianjin 300467, China shuai.chen_eo@outlook.com

**Keywords:** bound state in the continuum, Mie surface lattice resonances, multipoles, silicon metasurface, symmetry-protected

## Abstract

We propose and experimentally demonstrate a novel strategy to achieve dual-band symmetry-protected bound states in the continuum (BICs) in silicon metasurfaces. This strategy is based on the hybridization of Mie surface lattice resonances (SLRs) in periodic silicon bipartite nanodisk arrays, of which the central nanodisk displaced from the center of the unit cell. We show that dual-band electric quadrupole and magnetic dipole BICs can be supported in such a system, and transfer to quasi-BICs with ultrahigh measured quality factors up to 1240 at the Γ point. Taking advantage of the SLR characteristics, we show that the spectral separation and the quality factors of these two quasi-BICs can be conveniently tuned by varying the nanodisk diameter or the lattice period. Making use of these dual-band quasi-BICs, we numerically obtain bulk sensitivities above 480 nm/RIU and high figures of merit up to 1200. We also show that when the central nanodisk is not displaced but has different diameter, the silicon bipartite nanodisk array supports an electric dipole BIC that was referred to as subradiant SLR in the literature. Our work provides a new approach for realizing and tuning dual-band BICs, and the obtained ultrahigh-*Q* quasi-BICs can find potential applications in nonlinear optics, multimodal lasing, and optical sensing.

## 1 Introduction

Bound states in the continuum (BICs) are localized states coexisting with a continuous spectrum of radiating waves that can carry energy away [1]. Since the first experimental demonstration in 2013 [2], optical BICs have been attracting explosive attention in a wide range of photonic systems [1, 3], [4], [5]. As dark states with infinite radiative lifetime, BICs can provide a simple way to achieve very large quality factors (*Q* factors), and have emerged as an exciting platform for enhancing light–matter interactions [1, 3], [4], [5]. Over the years, BICs have found a diverse range of applications, including lasing with directional emission and ultralow threshold [6], [7], [8], enhanced nonlinear optical effects [9], [10], [11], [12], enhanced chiroptical responses [13], [14], [15], active photonics [16, 17], ultrasensitive sensing [18], [19], [20], and direct image differentiation [21].

Based on the physical mechanisms underlying the formation, BICs can be broadly classified into three types, and the most straightforward type is the symmetry-protected BIC formed due to the symmetry or separability restricted out-coupling [1, 3], [4], [5]. In periodic photonic structures such as gratings, metasurfaces, and photonic crystals, the symmetry-protected BICs occurring at Γ point have been widely studied. In order to transfer symmetry-protected BICs, which are completely decoupled from the radiating waves and are invisible, to quasi-BIC modes, which are accessible resonant modes with ultrahigh *Q* factors, one can either slightly break the excitation field symmetry through oblique incidences [2, 21, 22], or break the in-plane or out-of-plane structural symmetry under normal incidence [5]. The in-plane symmetry breaking is usually realized by deforming the structure of the meta-atom [23], [24], [25], or by introducing a relative tile between paired meta-atoms [14, 19, 26], whereas the out-of-plane symmetry breaking relies on disturbing the heights of a dimer or trimer cluster [27].

Recently, novel symmetry breaking approaches [28, 29] have also been explored. Among these, one exciting strategy is based on the hybridization of surface lattice resonances (SLRs). For example, Manjavacas and colleagues theoretically showed that in periodic plasmonic bipartite nanoparticle arrays, the electric dipole (ED) SLRs supported by two individual single-particle arrays can be cancelled due to destructive interference [30]. The resulted subradiant ED-SLR becomes a BIC when the two nanoparticles in the unit cell have the same size [31], as experimentally verified by Abujetas et al. in the terahertz regime [32]. However, only single-band ED-BICs were obtained, and the size difference between the two nanoparticles in the unit cell should be minimum (for example, ≤10% [31]) in order to achieve high quality factor for the quasi-BIC, posing challenges in nanofabrication and thus hindering the experimental demonstrations of high quality factors. Additionally, the advantages of the SLR-based strategy have not been mentioned.

In applications such as nonlinear frequency conversion, multimodal lasing, optical sensing, and multiplexed optical nanodevices, the existence of dual-band or even multiple resonances with high quality factors is beneficial [33]. Recently, dual-band BICs were theoretically reported in metasurfaces by shrinking or expanding the tetramerized holes of the superlattice [34], and were demonstrated with periodic asymmetric nano-holes [35], split rings [36], or nanodisks [37]. However, all these dual-band BICs were realized based on the destructive interference between adjacent antiphased localized resonances. To our knowledge, dual-band BICs based on the destructive interference of SLRs have not been reported yet.

Here we propose and experimentally demonstrate a novel strategy based on the hybridization of SLRs to achieve dual-band symmetry-protected BICs in metasurfaces. We will show that in periodic silicon bipartite nanodisk arrays, of which the in-plane structural symmetry is broken by displacing the central nanodisk from the center of the unit cell, the hybridization of Mie SLRs results in dual-band electric quadrupole (EQ) and magnetic dipole (MD) BICs at the Γ point. Taking advantages of the unique characteristics of the SLRs, we will further show that the spectral separation and the quality factors of these two quasi-BICs can be conveniently tuned by varying the nanodisk diameter or the lattice period. The potential applications of the obtained high-*Q* dual-band quasi-BICs will be illustrated with refractive index sensing. We will also show that the periodic silicon bipartite nanodisk array with the central nanodisk nondisplaced but having different diameter supports an ED-BIC, which was referred to as the subradiant SLR in [31].

## 2 Results and discussion

### 2.1 Spectra and fields of dual-band BICs

**Figure 1:**
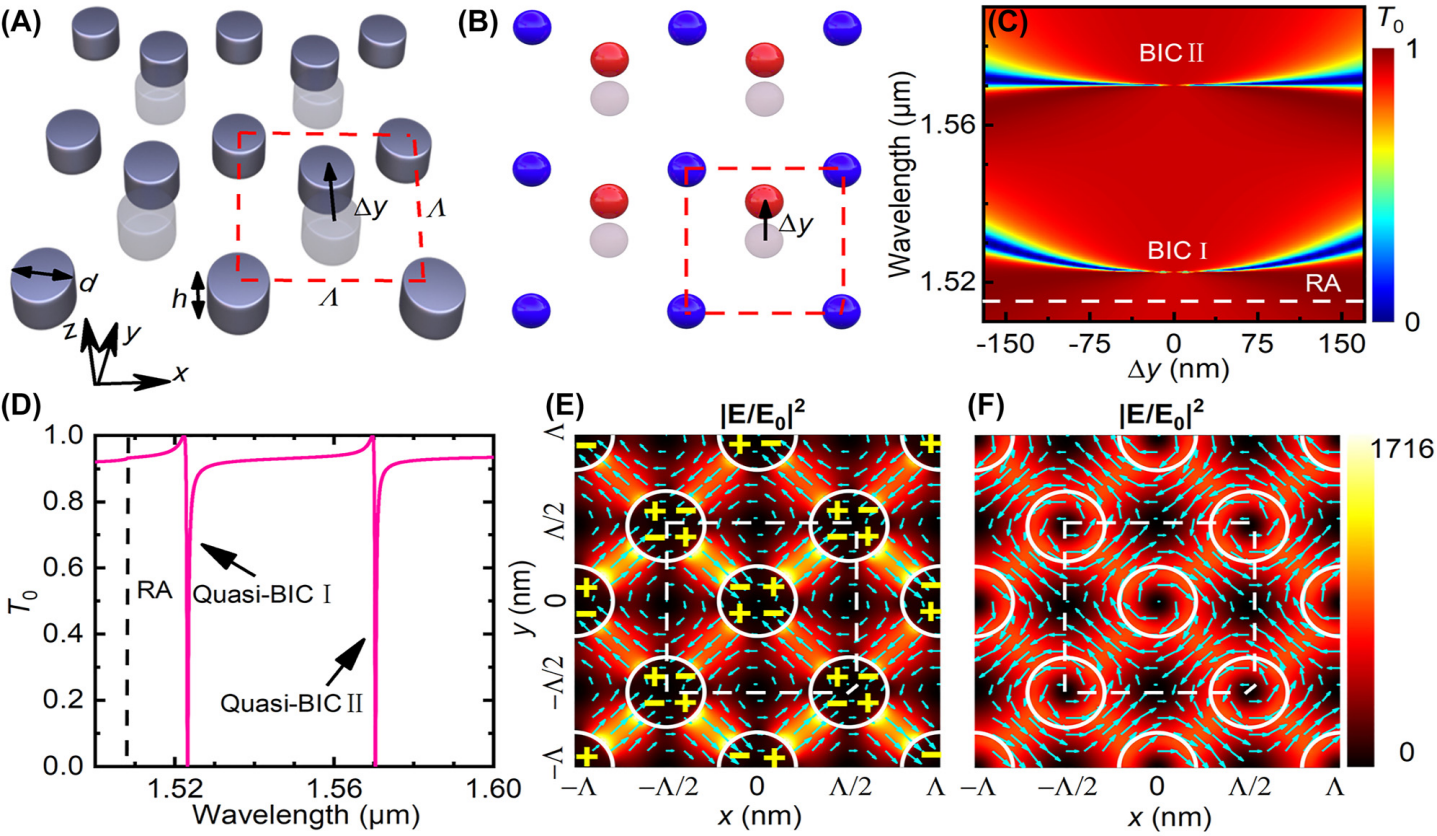
Proposed dual-band BICs in silicon bipartite nanodisk array. (A) 3D- and (B) top-viewed schematics of the silicon bipartite nanodisk array under study, which can be treated as the hybridization of two single-nanodisk arrays (in red and blue). The unit cell with square lattice of period Λ is illustrated by the red dashed box containing two silicon nanodisks with diameter *d* and thickness *h*. The central nanodisk is displaced vertically from the origin of the unit cell with Δ_*y*_. (C) and (D) Simulated zeroth-order transmittance spectra (C) as functions of Δ*y* or (D) for Δ*y* = 50 nm, showing two branches of extremely narrow dips. The dashed line indicates the (0, ±1) or (±1, 0) RA wavelength. (E) and (F) Near-field electric filed distributions (color for intensity and arrows for directions) in the *x* − *y* plane at the half height of silicon nanodisk for the resonance wavelengths of quasi-BIC I and II, as indicated by the dips in (D). Symbols “+” and “−” in (E) indicate charge distributions. The silicon nanodisks are outlined by white circles and a unit cell is outlined by the dashed box in (E) and (F).

Figure 1A and B illustrate the silicon bipartite nanodisk array under study. This array can be treated as the hybridization of two single-nanodisk arrays, which are indicated by different colors in Figure 1B. The unit cell of square lattice with periodicity Λ is composed of two silicon nanodisks with the same diameter *d* and height *h* = 100 nm. The central nanodisk is displaced from the center of the unit cell with a distance Δ*y* in the *y* direction. The nanodisks are embedded in homogeneous surrounding environment with refractive index *n*_0_ = 1.45. The silicon metasurface is normally illuminated by plane wave with electric field polarized along the *x* axis and of unitary amplitude (|*E*_0_| = 1).

We adopted a home-built package based on rigorous coupled-wave analysis developed following [38], [39], [40] to model the zeroth-order transmittance spectra and the near-field distributions of the silicon metasurface. In all the simulations, the wavelength-dependent refractive indices of silicon were taken from [41]. Unless otherwise specified, the simulations were performed with Λ = 1040 nm and *d* = 440 nm.

Figure 1C depicts the simulated zeroth-order transmittance spectra for different displacements Δ*y*. Results show that there exist two branches of Fano-shaped transmittance dips locating near the RA line of the (0, ±1) or the (±1, 0) order, which is expressed as

(1) $$\lambda_{\text{RA}}=n_{0}\Lambda .$$

Figure 1D presents a typical zeroth-order transmittance spectra for Δ*y* = 50 nm, in which the two transmittance dips have Fano lineshape. These two dips become narrower as |Δ*y*| decreases, and completely disappear when Δ*y* = 0. The symmetric patterns relative to the axis of Δ*y* = 0 and the disappearance of resonances when Δ*y* = 0 suggest the occurrence of dual-band symmetry-protected BICs. We assign these two BICs operating at the shorter and longer wavelengths as BIC I and BIC II, respectively. For Δ*y* ≠ 0 both BICs transit into quasi-BICs with observable spectral features and finite linewidths. The resonance wavelengths of the quasi-BIC I and II for Δ*y* = 50 nm are *λ*_qBIC_ = 1.523 μm and 1.570 μm, respectively, as indicated by the transmittance dips of Figure 1D, and the corresponding linewidths are extracted (Figure S1 in the Supplementary) to be *δλ* = 0.49 nm and 0.38 nm, respectively. Therefore, the numerical quality factors, determined by *Q* = *λ*_qBIC_/*δλ*, are calculated to be *Q* = 3069 and 4071 for the quasi-BIC I and II, respectively. Figure 1E and F present the near-field electric field distributions in the *x* − *y* plane at the half height of silicon nanodisk for these two quasi-BICs when Δ*y* = 50 nm. For the quais-BIC I, Figure 1E shows that the electric field confined between neighboring nanodisks forms two pairs of electric quadrupoles within a unit cell, which have the opposite directions for two neighboring rows or columns. Because of the structural asymmetry with respect to the *x* axis, the strength of the top electric quadrupole due to a smaller inter-nanodisk distance is stronger than that of the bottom one. This leads to partial cancellation of these two electric quadrupoles, which provides a radiation channel to the BIC, forming a quasi-EQ-BIC.

For the quasi-BIC II, Figure 1F shows that the electric fields are circulating around the silicon nanodisks in clockwise and counterclockwise directions for two neighboring rows or columns, respectively, forming two magnetic dipoles with opposite directions within a unit cell. The broken symmetry results in partial cancellation of these two magnetic dipoles, forming a quasi-MD-BIC.

### 2.2 Multipole decomposition

In order to further understand the nature of these two BICs, we employ the multipole decomposition approach to reveal the physical mechanisms underlying the formations of these BICs in terms of multipolar modes. We consider multipolar modes up to quadrupole modes. The scattered powers of the ED, MD, EQ, and magnetic quadrupole (MQ) moments can be calculated with [42, 43]

(2) $$I_{P}=\frac{2\omega^{4}}{3c^{3}}|\vec{P}|,$$

(3) $$I_{M}=\frac{2\omega^{4}}{3c^{3}}|\vec{M}|,$$

(4) $$I_{Q^{\left(e\right)}}=\frac{\omega^{6}}{5c^{5}}\sum |\overset{\left(e\right)}{\underset{\alpha ,\beta }{\vec{Q}}}|,$$

(5) $$I_{Q^{\left(m\right)}}=\frac{\omega^{6}}{40c^{5}}\sum |\overset{\left(m\right)}{\underset{\alpha ,\beta }{\vec{Q}}}|,$$

respectively. Here *c* is the speed of light, *ω* is the frequency of light. The multipole moments can be defined as [44]

(6) $$\vec{P}=\frac{1}{i\omega }\int \vec{J}d^{3}r,$$

(7) $$\vec{M}=\frac{1}{2c}\int \vec{r}\times \vec{J}d^{3}r,$$

(8) $${\vec{Q}}_{\alpha ,\beta }^{\left(e\right)}=\frac{1}{2i\omega }\int \left[r_{\alpha }J_{\beta }+r_{\beta }J_{\alpha }-\frac{2}{3}\left(\vec{r}\cdot \vec{J}\right)\delta_{\alpha ,\beta }\right]d^{3}r,$$

(9) $${\vec{Q}}_{\alpha ,\beta }^{\left(m\right)}=\frac{1}{3c}\int \left[{\left(\vec{r}\cdot \vec{J}\right)}_{\alpha }r_{\beta }+{\left(\vec{r}\cdot \vec{J}\right)}_{\beta }r_{\alpha }\right]d^{3}r$$

where *α*, *β* = *x*, *y*, *z*, and the current density distribution in a unit cell can be calculated with $\vec{J}=-i\omega \epsilon_{0}\left(n^{2}-1\right)\vec{E}$ .

**Figure 2:**
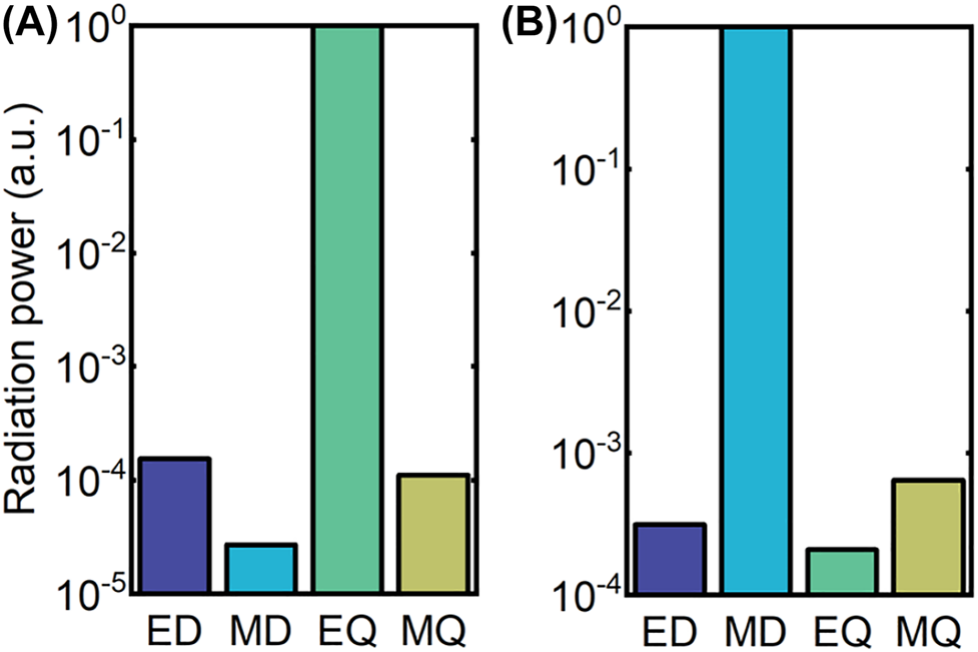
Multipolar contents of (A) quasi-BIC I and (B) quasi-BIC II for Δ*y* = 50 nm. The vertical axis shows normalized radiation powers of electric and magnetic dipole (ED and MD) moments, and electric and magnetic quadrupole (EQ and MQ) moments.

Figure 2 shows that, for the quasi-BIC I formed when Δ*y* = 50 nm, the dominant contributing multipolar mode is the EQ, whereas that for the quasi-BIC II, the dominant mode is the MD. These results are consistent with the near-field distributions as shown in Figure 1E and F, respectively. Therefore, the near-field distributions and the multipolar contributions have revealed that, the BIC I and II are an EQ-BIC and an MD-BIC, respectively.

Note that for other displacements, the simulated transmittance spectra and the near-field electric field distributions are similar. As a result, the multipole decomposition leads to the same conclusions (Figure S2 in the Supplementary). In other words, the underlying physics of these two quasi-BICs does not change as Δ*y* varies.

### 2.3 Experimental validations of dual-band quasi-BICs

**Figure 3:**
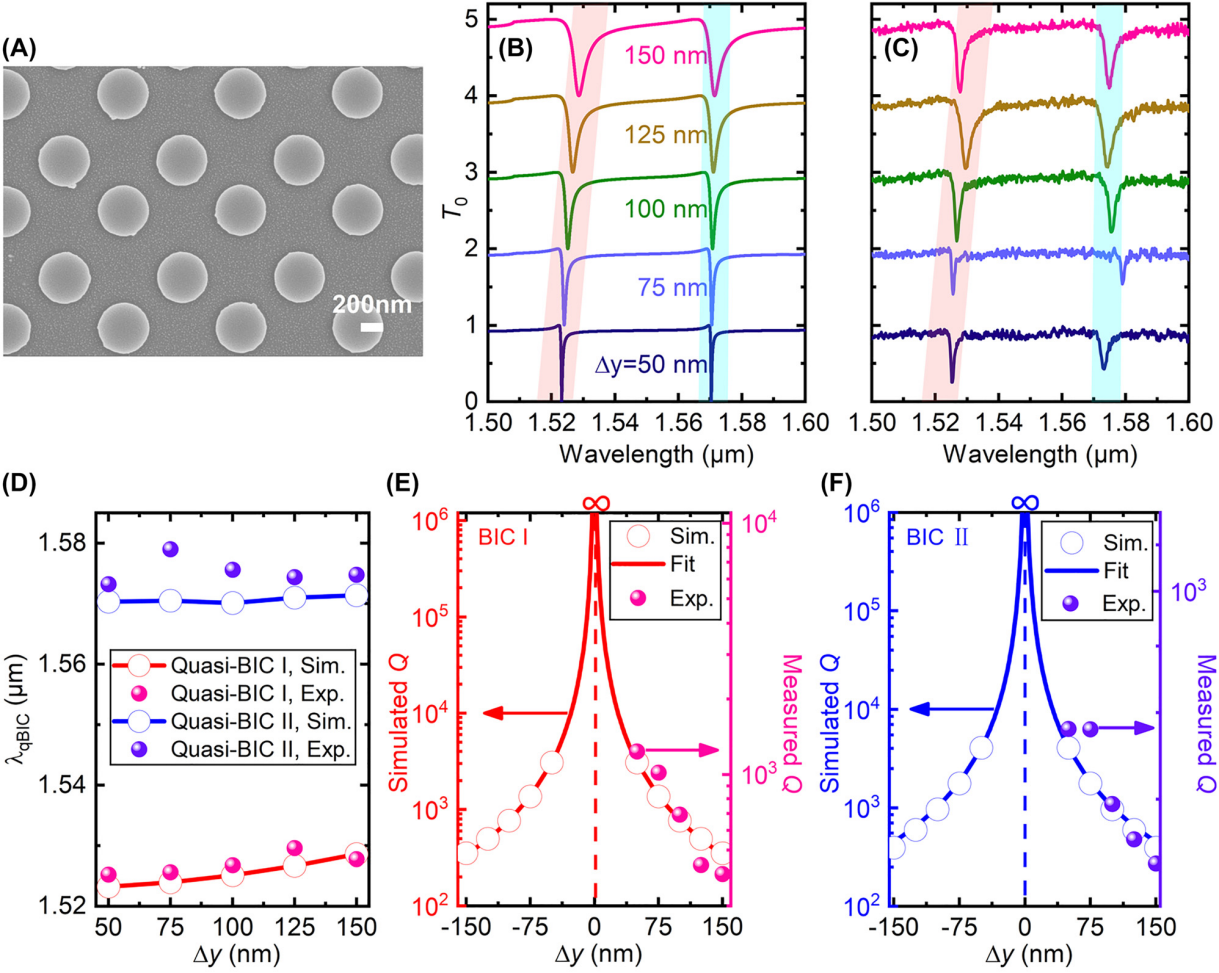
Experimental demonstration of the dual-band quasi-BICs. (A) SEM image of a typical as-fabricated sample with Δ*y* = 50 nm. (B) Simulated and (C) measured zeroth-order transmittance spectra of silicon bipartite nanodisk array with different displacements. (D)–(F) Comparison of simulation (circles) and experimental (balls) results on (D) extracted resonance wavelengths and, (E) and (F) quality factors of the quasi-BICs as functions of Δ*y*. Solid curves in (E) and (F) denote theoretical fitting with an inverse square function.

To verify our theoretical findings, we fabricated silicon bipartite nanodisk arrays on a quartz substrate using the state-of-art nanofabrication techniques. We first deposited a 100 nm thick film of amorphous silicon on the substrate using plasma enhanced chemical vapor deposition. A thin film of electron beam resist (ZEP 520A) was then spin-coated on the top, and patterned with electron-beam lithography. A 40 nm film of chromium was deposited using the electron-beam evaporation, followed by liftoff process. The obtained chromium nanodisks pattern were then transferred to the silicon using inductively coupled plasma-reactive ion etching. Finally, the chromium mask was removed by wet etching. The as-fabricated samples have a large area of 1.2 mm × 1.2 mm, which contains a sufficiently large number of nanodisks for achieving ultrahigh quality factors in experiments [45]. The scanning electron image of a typical as-fabricated sample is shown in Figure 3A.

The as-fabricated samples were then covered with a quartz glass using index-matching oil in order to guarantee homogeneous dielectric environment surrounding the silicon nanodisks. The sample mounted on a 3D linear translation stage is normally illuminated by collimated white light, which is from a 100 W halogen source (HL100, Ideaoptics), has beam diameter of 1 mm, and is polarized along the *x* axis. The transmitted beams were collected by a high-performance optical spectrum analyzer (AQ6380, Yokogawa) using a lens-based fiber-coupling system.

Figure 3B and C compare the simulated and measured zeroth-order transmittance spectra for different displacements Δ*y*. Results show that in general the measured transmittance spectra agree well with the simulation results. As Δ*y* increases from 50 nm to 150 nm in step of 25 nm, the resonance wavelength of the quasi-BIC I is slightly red-shifted, whereas that of the quasi-BIC II experiences minimum red-shift, as better visualized by Figure 3D. On the other hand, as Δ*y* decreases, the quality factors extracted from the simulation and experimental spectra also agree well for both quasi-BICs, as shown by Figure 3E and F. For the quasi-BIC I, the measured quality factors (Figure S1 in the Supplementary) are about 40% of the simulated results, and reach as high as *Q* = 1240 for Δ*y* = 50 nm. As a comparison, the measured quality factors are about 16% of the simulated results for the quasi-BIC II, and can reach *Q* = 630 for Δ*y* = 50 nm.

Remarkably, as Δ*y* approaches zero, both the simulated and measured quality factors increase significantly and tend to infinity. For relatively small displacements, the dependence of these quality factors on the asymmetry parameter Δ*y* is inverse quadratic for both quasi-BICs, as shown by the fitted curves, which follow

(10) $$Q\propto 1/{\left(\Delta y\right)}^{2}.$$

This inverse square relation is consistent with the universal law for symmetry-protected quasi-BICs [23]. Therefore, we have theoretically and experimentally validated the symmetry-protected characteristics of the dual-band BICs.

In Figure 3B–F, we also find that when compared with the quasi-BIC II, the quasi-BIC I has better agreement between the experimental and simulation results, in terms of the spectra, the resonance wavelengths, and the estimated quality factors. This can be explained by their different near-field electric field distributions: the electric fields of the quasi-BIC I are mainly confined to the spaces between neighboring nanodisks, whereas a large portion of the electric fields are confined within the nanodisks for the quasi-BIC II. As a consequence, the quasi-BIC II suffers from more losses, including more absorption loss of the deposited amorphous silicon, and more scattering losses due to the inevitable fabrication imperfections such as variations in shapes and sizes, and side-wall tilting.

### 2.4 Tuning via the nanodisk diameter

**Figure 4:**
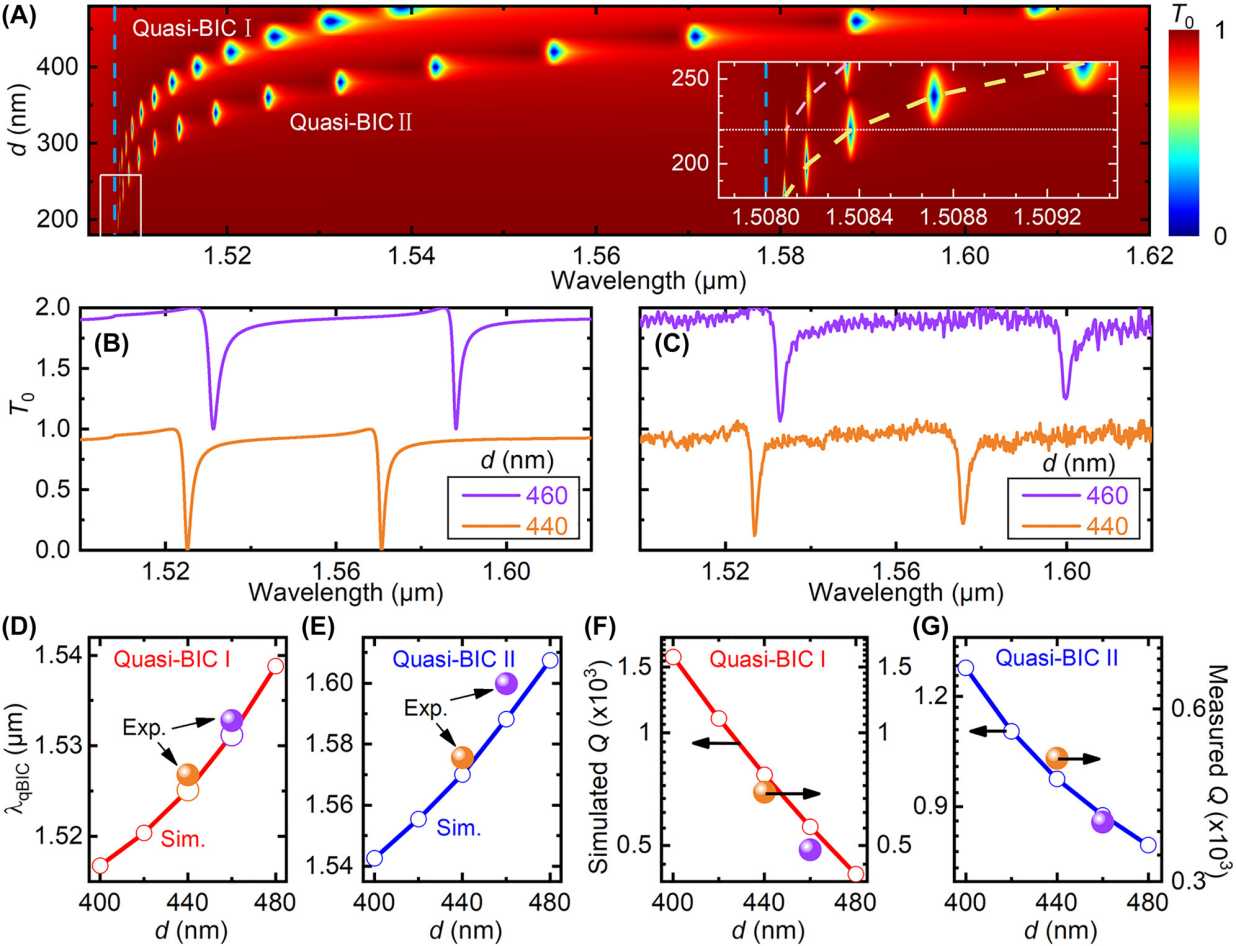
Tuning of the dual-band quasi-BICs via nanodisk diameter. (A) and (B) Simulated, and (C) measured zeroth-order transmittance spectra of silicon bipartite nanodisk array with different diameters. Inset in (A) is a zoom-in of the white dashed box, showing the truncation of the quasi-BIC I when *d* < 220 nm, as shown by the horizontal white dotted line. The blue vertical dashed line in (A) indicates *λ*_RA_. The dashed curve in (A) inset provides a guide to the eyes. (D)–(G) Comparison of simulation (circles) and experimental (balls) results on (D) and (E) extracted resonance wavelengths and (F) and (G) quality factors of quasi-BIC I and II as functions of *d*.

While until now we fixed the nanodisk diameter *d* to be 440 nm, here we will show that the resonance wavelengths and the quality factors of these two quasi-BICs can be further tuned conveniently by varying the nanodisk diameter. Figure 4A shows that as *d* decreases from 480 nm to 200 nm, the resonance wavelengths of the quasi-BIC I and II are both blue-shifted, but to different extends: the quasi-BIC I experiences much smaller blue-shifts than the quasi-BIC II. In other words, the spectral separation between the quasi-BIC I and II decreases with *d*. Indeed, as the nanodisk diameter decreases the resonance wavelengths of both quasi-BICs converge to the (0, ±1) or (±1, 0) RA wavelength, which is indicated by the vertical dashed line. On the other hand, the smaller the nanodisk diameter, the smaller the linewidth and thus the larger the quality factor for both quasi-BICs. These behaviors are consistent with those of the SLRs [45, 46].

For small nanodisk diameters of *d* = 240 nm and 220 nm, Figure 4A shows that the quasi-BIC I has shallow transmittance dips, suggesting relatively weak resonances. In these scenarios, the spectral separations between the two quasi-BICs are 0.54 nm and 0.27 nm, respectively. As *d* further decreases, the quasi-BIC I is truncated. This is because a too small silicon nanodisk cannot support the electric quadrupole any more. In other words, the silicon nanodisk diameter should be large enough in order to support the EQ-BIC.

To validate these theoretical results, we fabricated two samples with nanodisk diameters of *d* = 440 nm and 460 nm, respectively. Comparing Figure 4B and C we find that, in general the measured transmittance spectra agree well with the simulation results. We extract the resonance wavelengths and the quality factors from the measured transmittance spectra, and plot them together with the simulation results. Figure 4D–G show that the quasi-BIC I has better agreement between the simulation and experimental results over the quasi-BIC II, in terms of the resonance wavelength and the quality factor. The reason can be explained similar to the behaviors as functions of Δ*y* in Figure 3D–F.

Note that Figure 4E has larger *Y*-axis scale than Figure 4D, since the resonance wavelength of the quasi-BIC II experiences larger blue-shifts as *d* decreases. On the other hand, Figure 4G has smaller *Y*-axis scale than Figure 4F, because the quality factor of the quasi-BIC II is always slightly lower than that of the quasi-BIC I. This is consistent with the smaller electric field enhancement of the quasi-BIC II than the quasi-BIC I: when Δ*y* = 50 nm the maximum electric field enhancement is only 870 for the former but reaches |*E*/*E*_0_|^2^ = 1716 for the latter, as shown by Figure 1E and D, respectively.

### 2.5 Tuning via the lattice period

**Figure 5:**
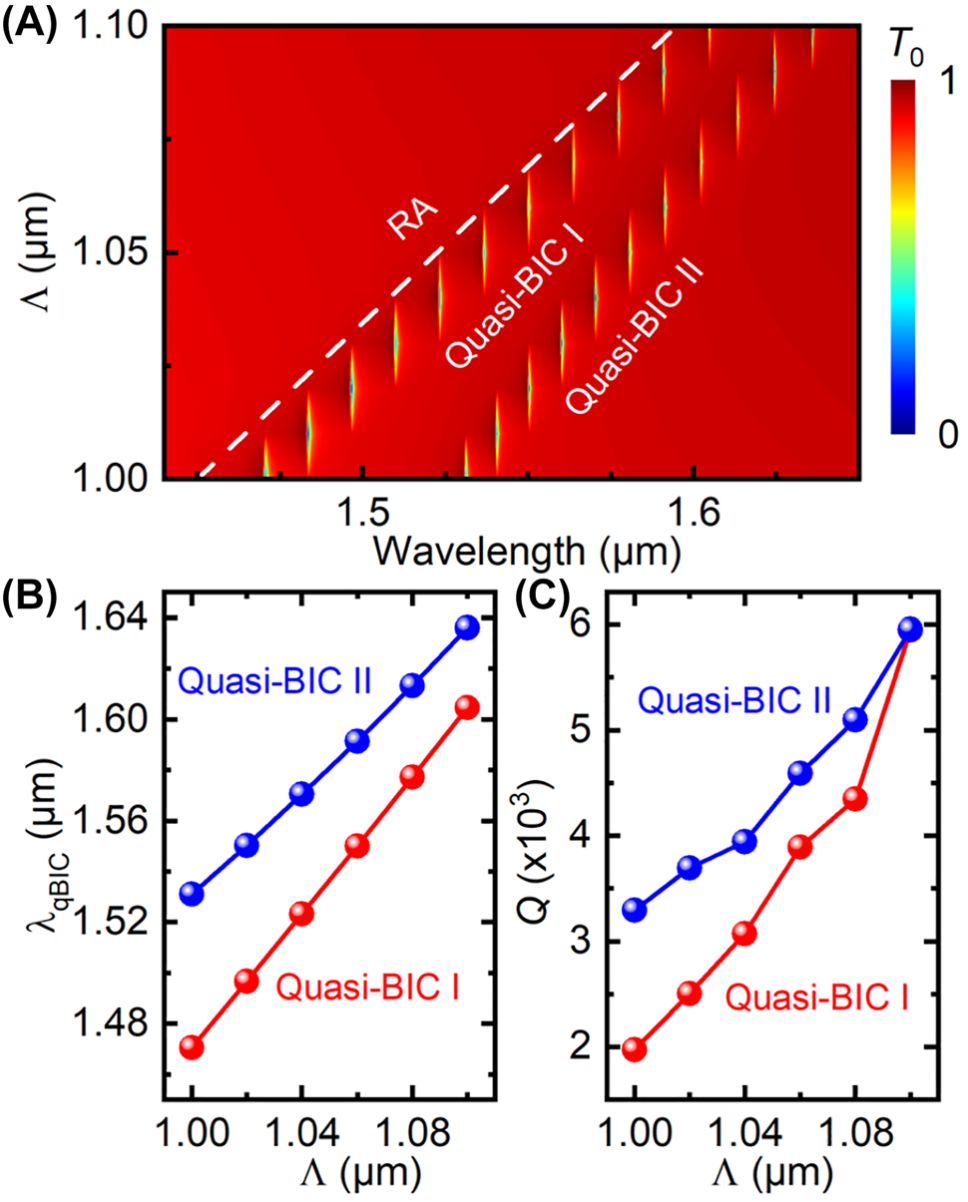
Tuning of the dual-band quasi-BICs via lattice period. (A) Simulated zeroth-order transmittance spectra for different lattice periods. The white dashed line indicates the (0, ±1) or (±1, 0) RA line. (B) Extracted resonance wavelengths and (C) quality factors as functions of Λ.

Taking advantage of the tunable characteristics of the SLRs, we can also conveniently tune the dual-band quasi-BICs by changing the lattice period Λ. Figure 5A shows that as Λ increases from 1 μm to 1.1 μm, both quasi-BICs are red-shifted and approach the (0, ±1) or (±1, 0) RA line, which is indicated by the white dashed line. This behavior is better visualized by Figure 5B, which shows that the resonance wavelengths of the quasi-BIC I and II increase linearly with Λ. On the other hand, the quality factors of both quasi-BICs increase exponentially with Λ, as shown by Figure 5C, which is in a logarithmic scale.

The tunable characteristics of the dual-band quasi-BICs on the resonance wavelengths and the quality factors are consistent with those of the SLRs [46] and thus can be explained similarly: as Λ increases, the delocalized nature of the resonances become more important, resulting in smaller separation from the RA line and higher quality factors.

### 2.6 Refractive index sensing application

The ultra-narrow linewidths and the significant field enhancement make the dual-band quasi-BICs ideal platforms for sensing applications. As a specific example, here we numerically investigate the refractive index sensing.

**Figure 6:**
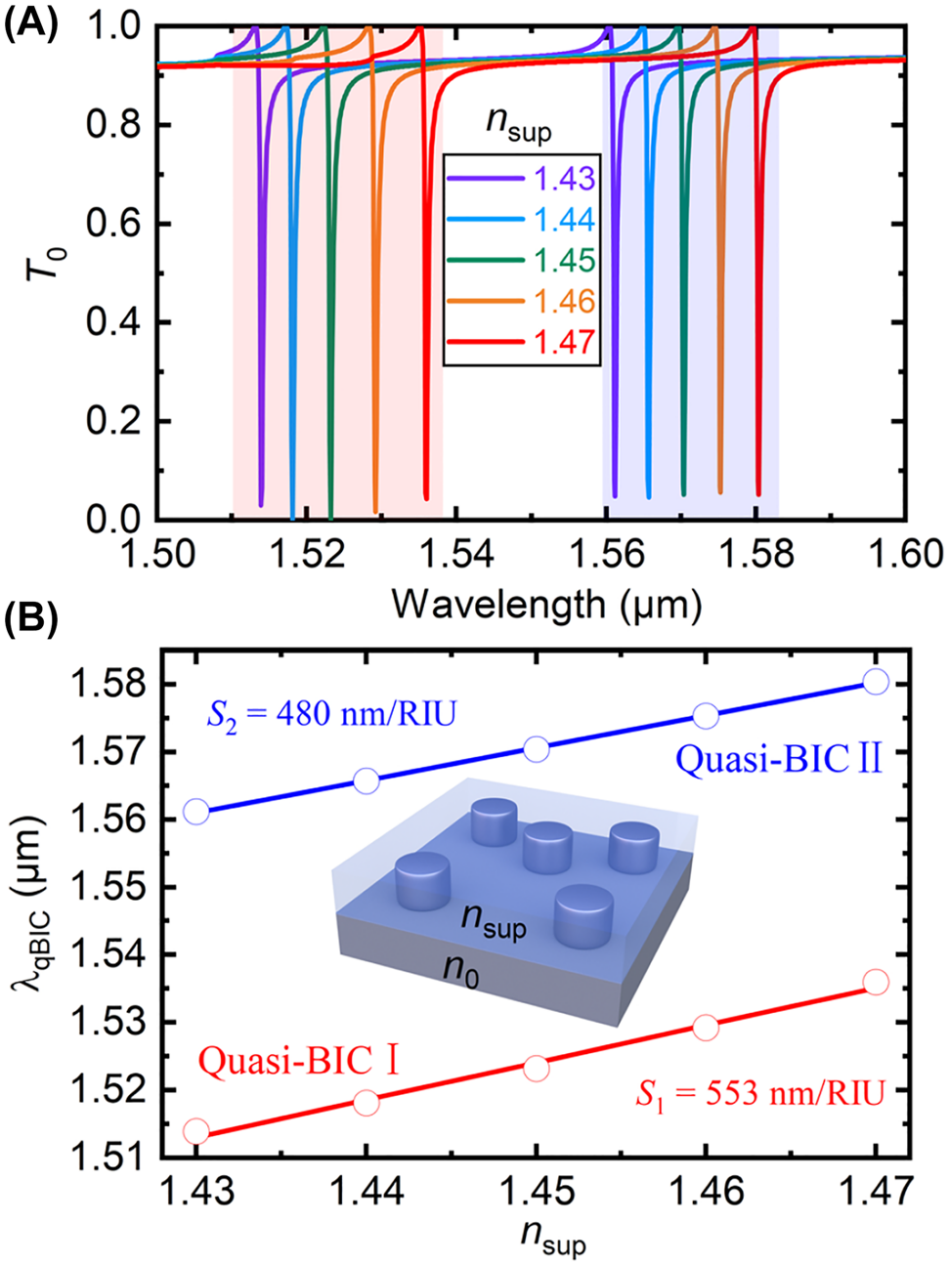
Refractive index sensing performance of the dual-band quasi-BICs. (A) Simulated zeroth-order transmittance spectra for different superstrate refractive indices. (B) Extracted resonance wavelengths as a function of *n*_sup_. Inset illustrates the refractive index sensing configuration based on the periodic silicon bipartite nanodisks with dual-band quasi-BICs.

Figure 6A depicts the simulated transmittance spectra of the periodic silicon bipartite nanodisks with different superstrate refractive indices *n*_sup_, of which the unit cell is illustrated by Figure 6B inset. Simulation results show that as *n*_sup_ increases from 1.43 to 1.47, the resonance wavelengths of both quasi-BICs are red-shifted. The bulk sensitivities, which can be determined by *S* = Δ*λ*_qBIC_/Δ*n*_sup_, are calculated to be *S*_1_ = 553 nm/RIU and *S*_2_ = 480 nm/RIU for the quasi-BIC I and II, respectively, as shown by Figure 6B. We then obtained the corresponding figures of merit (FOMs), defined by FOM ≡ *S*/*δλ*: FOM_1_ = 1106 and FOM_2_ = 1200 for the quasi-BIC I and II, respectively. Strikingly, the obtained bulk sensitivities and FOMs outperform many sensors based on other quasi-BICs [18, 19]. The superior sensing performance can attribute to the significant field enhancement extending over large volumes outside the silicon nanodisks, and the extremely narrow linewidths.

### 2.7 Nondisplaced central nanodisk with different diameters

**Figure 7:**
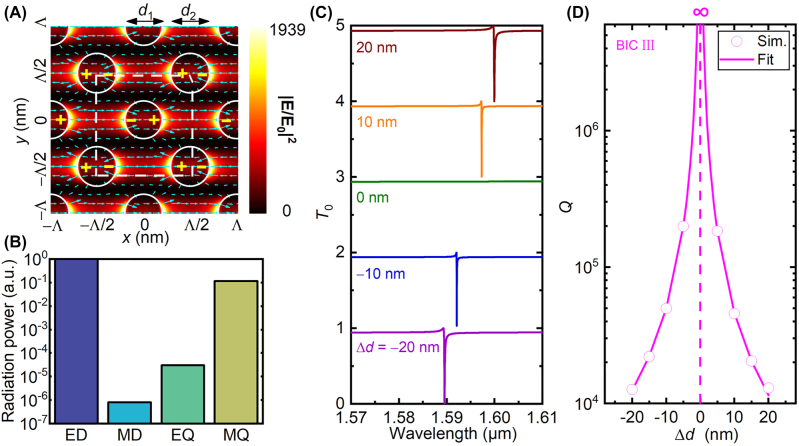
Another BIC in silicon bipartite nanodisk array with the central nanodisk nondisplaced but of different diameter. (A) Near-field electric filed distributions (color for intensity and arrows for directions) in the *x* − *y* plane at the half height of silicon nanodisk and (B) multipolar contributions for the quasi-BIC III, as indicated by the transmittance dip for Δ*d* = −20 nm in (C). Symbols “+” and “−” in (A) indicate charge distributions. The silicon nanodisks are outlined by white circles, and the unit cell is outlined by the dashed box, showing the silicon bipartite nanodisk array with the central nanodisk nondisplaced but having different diameter. Δ*d* = *d*_1_ − *d*_2_ is the diameter difference between the central and corner nanodisks within the unit cell. (C) Simulated zeroth-order transmittance spectra of silicon bipartite nanodisk array for different diameter differences. (D) Extracted quality factors of quasi-BIC III as functions of Δ*d*.

Finally, we investigate the silicon bipartite nanodisk array with the central nanodisk nondisplaced but having diameter different from corner nanodisks in the unit cell, as outlined by the dashed box in Figure 7A. This metasurface is similar to the one studied in [31], except that gold is replaced by silicon here. The asymmetry degree is now described by the diameter difference, Δ*d* = *d*_1_ − *d*_2_, where *d*_1_ and *d*_2_ are the diameters of the central and corner nanodisks of the unit cell, respectively. Figure 7C depicts the simulated zeroth-order transmittance spectra as Δ*d* varies from −20 nm to 20 nm in step of 10 nm, where *d*_1_ = 440 nm is adopted, the same diameter used in our previous discussion. Results show that, there exist only one transmittance dip for nonzero Δ*d*, and that it becomes narrower as |Δ*d*| decreases. When Δ*d* = 0 nm (the green curve), the transmittance completely disappears. The extracted quality factors are shown in Figure 7D. It is evident that the dependence of the quality factor on the asymmetry parameter Δ*y* is also inverse quadratic, as shown by the curve fitted with

(11) $$Q\propto 1/{\left(\Delta d\right)}^{2}.$$

These characteristics suggest the occurrence of another symmetry-protected BIC (denoted as BIC III) in the silicon bipartite nanodisk array, which can be accessed in the form of quasi-BIC through varying the central nanodisk diameter. In order to understand the underlying physics, in Figure 7A we plot the near-field electric field distributions in the *x* − *y* plane at the half height of silicon nanodisk for the quasi-BIC III when Δ*d* = −20 nm. Results show that Mie ED-SLR is excited in these silicon nanodisks, and that the electric dipoles induced in neighboring rows of nanodisks have opposite directions due to the opposite phase. As a result, the partial cancellation of these ED-SLRs due to different diameters leads to the quasi-BIC III. Specially, when Δ*d* = 0, the cancellation is perfect, corresponding to the occurrence of the BIC III.

We also employed the multipole decomposition approach to further unveil the physical mechanism of the formation of the BIC III. Figure 7B shows that the dominant contributing multipolar mode is the ED. In other words, the BIC III is an ED-BIC. This conclusion is consistent with the near field distribution shown in Figure 7A.

The spectra behaviors and the near field distributions are similar to the gold bipartite nanodisk array with central nanodisk having different diameter [30, 31]. Therefore, we do not perform experimental validations in this work. The difference is that Mie ED-SLRs are excited in all-dielectric silicon nanodisks, whereas plasmonic ED-SLRs are excited in gold nanodisks [30, 31]. Compared with [30, 31], here we further showed that the quality factor obeys an inverse square dependence on Δ*d*, confirming theoretically that the so-called subradiant SLR in [31] is a symmetry-protected BIC, and that the multipole decomposition results further point to an ED-BIC.

## 3 Concluding remarks

In conclusions, we have proposed and experimentally demonstrated a novel strategy to realize dual-band symmetry-protected BICs based on the hybridization of SLRs in periodic silicon bipartite nanodisk arrays, of which the central nanodisk is displaced from the center of the unit cell. The near-field distributions and the multipole decomposition results have suggested that an EQ-BIC and an MD-BIC can be excited slightly above the (0, ±1) or (±1, 0) RA wavelength. By taking advantage of the tuning characteristics of the SLRs, we have shown that the resonance wavelengths and the quality factors of both quasi-BICs can be conveniently tuned by varying the nanodisk diameter or the lattice period. Experimental data have provided good agreement with simulation results. Remarkably, we have experimentally demonstrated dual-band quasi-BICs with ultrahigh *Q* factors of 1240 and 630, respectively. We expect that the measured quality factors can be further improved significantly by fabricating the samples based on silicon-on-insulator (SOI) wafers, which can inhibit the absorption loss of the as-deposited amorphous silicon, as suggested in [10, 12]. Making use of the ultra-narrow linewidths and the significant electric field enhancements of the dual-band quasi-BICs, we have theoretically shown that the bulk sensitivities can reach 553 nm/RIu and 480 nm/RIU, and that the corresponding FOMs are 1106 and 1200 for the quasi-BIC I and II, respectively. We have also numerically shown that the silicon bipartite nanodisk array with the central nanodisk nondisplaced but having different diameter can support an ED-BIC, which was referred to as the subradiant SLR in [31]. We therefore expect the silicon bipartite nanodisk arrays supporting dual-band EQ- and MD-BICs, or a single-band ED-BIC, depending on the symmetry breaking scheme, can serve as an attractive metasurface platform for applications in nonlinear optical frequency conversion, low-threshold lasing, and chemical or biological sensing, etc. As a final remark, we mention that since the central nanodisk of the unit cell can be displaced independently along the *x* and *y* axes, the proposed strategy holds the promise for further realizing the polarization-controlled BICs [47, 48], which will be studied in the future.

## Supplementary Material

Supplementary Material Details
