# Supplementary material for: Dual-band bound states in the continuum based on hybridization of surface lattice resonances
Source: Nanophotonics. 2022 Nov 1;11(21):4843–53. doi: 10.1515/nanoph-2022-0427 (PMC11501325; doi:10.1515/nanoph-2022-0427)
Supplement: Supplementary file 1 — Supplementary Material Details [file j_nanoph-2022-0427_suppl.docx]

**Supplementary Material**

**Dual-band bound states in the continuum based on hybridization of lattice resonances**

Xiang Du, Lei Xiong, Xueqian Zhao, Shuai Chen, Jianping Shi*, and Guangyuan Li*

**
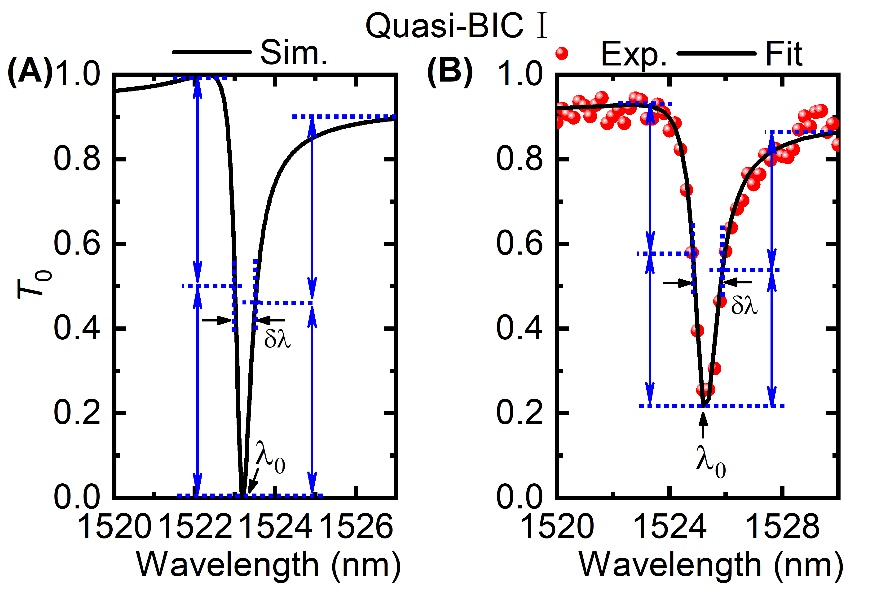
**

**Figure S1.** Illustration of the extraction processes of the linewidths and quality factors from (A) simulated and (B) measured transmittance spectra. This is exemplified by the transmittance spectra of the quasi-BIC I when Δy=50 nm. For the simulated transmittance spectra, the extraction processes are as follows [1]: we first determine two baselines for the left-hand and the right-hand sides, find the location of the transmittance dip, and then calculate the wavelengths for the two half-maximums between the left-hand baseline and the dip value, and between the right-hand baseline and the dip value. The width between these two wavelengths at half-maximums is estimated to be the linewidth δλ. With the dip wavelength λ_0_ and the linewidth δλ, the quality factor is then calculated with Q = λ_0_/δλ. For the experimental transmittance spectra, a Fano fit is first performed [2], and we then extract the resonance wavelength, the linewidth, and the quality factor from the fitted curve.


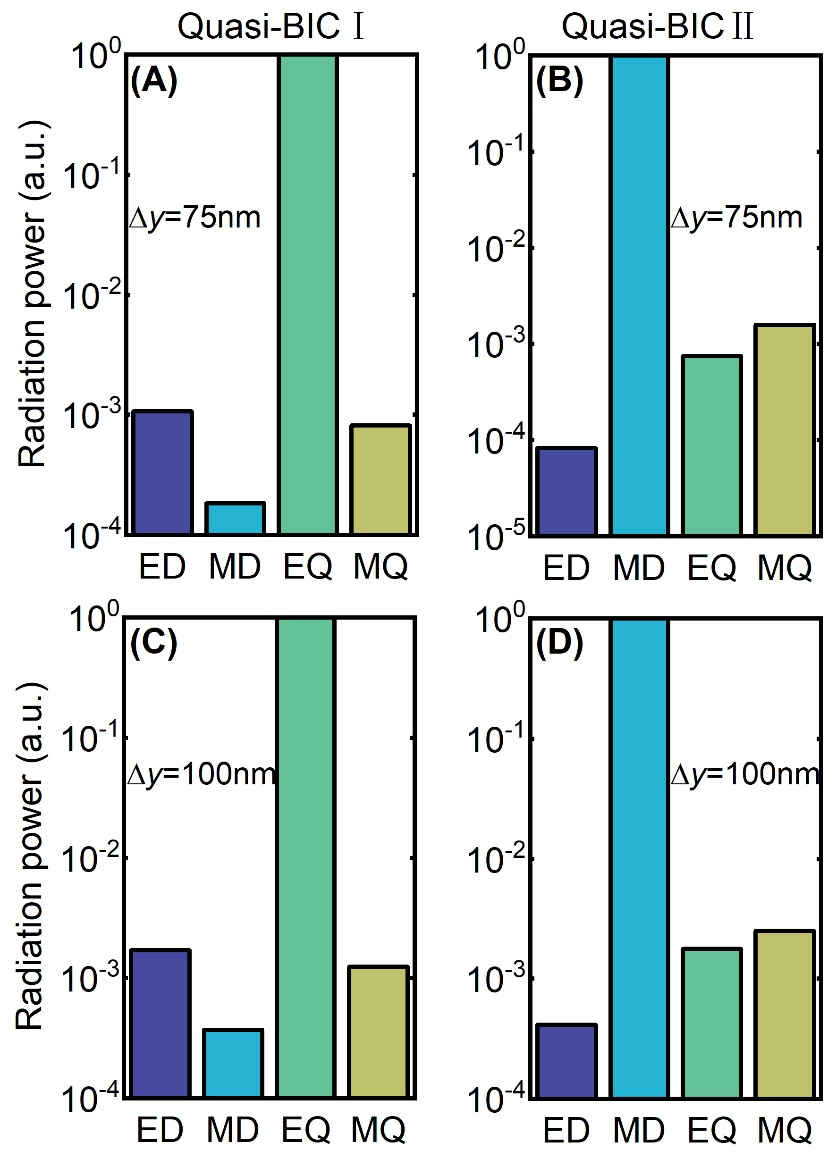


**Figure S2.** Multipolar contents of (A)(C) quasi-BIC I and (B)(D) quasi-BIC II for (A)(B) Δy=75 nm and (C)(D) Δy=100 nm. The vertical axis shows normalized radiation powers of electric and magnetic dipole (ED and MD) moments, and electric and magnetic quadrupole (EQ and MQ) moments. Results show that the quasi-BIC I is an EQ-BIC, and the quasi-BIC II is an MD-BIC, regardless of the displacement.

**Reference:**

[1] Supplementary Material of X. Fang, L. Xiong, J. Shi, and G. Li. "High-*Q* quadrupolar plasmonic lattice resonances in horizontal metal-insulator-metal gratings". *Opt. Lett.* vol. 46, 2021, pp. 1546-1549. <https://doi.org/10.1364/OL.419364>

[2] Supplementary Material of M. S. Bin-Alam, O. Reshef, Y. Mamchur, et al., "Ultra-high-𝑄 resonances in plasmonic metasurfaces," *Nat. Commun.*, vol. 12, 2021, pp. 974. <https://doi.org/10.1038/s41467-021-21196-2>
